# Angiome géant de la face

**DOI:** 10.11604/pamj.2021.39.121.28860

**Published:** 2021-06-11

**Authors:** Zahra Sayad, Malik Boulaadas

**Affiliations:** 1 Department of Oral and Maxillofacial Surgery, Ibn Sina University Hospital Center, Rabat, Morocco

**Keywords:** angiome, géant, face, angioma, giant, facial

## Abstract

We here report the case of a 78 year old woman with no previous history, whose medical history only included facial angioma since birth. The patient had never consulted a doctor before. Twelve months before, hypertrophy of the left jugo-naso-labial soft tissues causing lip incontinence with feeding and pronunciation difficulty as well as asymmetry with lack of facial expression occurred. Clinical examination showed red-violet jugo-naso-labial swelling, not exceeding the frontal, nasal and upper labial median line, extending to the tragus, with nodosities at the level of the upper half of the left side wall of the nose and the upper part of the cheek deviating the nose to the right side. It also showed voluminous hypertrophy of the entire lower lip that descended downwards, masking the chin and the upper part of the neck and causing incomplete closure of the mouth (A). The patient underwent reduction surgery under general anesthesia for aesthetic and, above all, functional purposes. Then she underwent two surgical tweaks under local anesthesia (B). The patient was very satisfied with functional and aesthetic results. The patient had a 3-year follow-up with no recurrences.

## Image en médecine

Nous rapportons le cas d´une femme âgée de 78 ans, sans antécédents particuliers, qui présente depuis la naissance, un angiome plan de toute l´hémiface gauche. A noter que la patiente n´a jamais consulté auparavant. L´évolution fut marquée il y a 12 mois, par une hypertrophie des parties molles jugo-naso-labiales gauche occasionnant une incontinence labiale avec une difficulté d´alimentation et de prononciation, une asymétrie avec défaut d´expression faciale. L'examen clinique montre une tuméfaction jugo-naso-labiale, de couleur rouge-violacée ne dépassant pas la ligne médiane au niveau frontal, nasal et labial supérieur, arrivant jusqu´au tragus, avec des nodosités au niveau de la moitié supérieure de la paroi latérale gauche du nez et la partie supérieure de la joue déviant le nez vers le côté droit. On note aussi une hypertrophie volumineuse de toute la lèvre inférieure qui descend vers le bas masquant le menton et la partie supérieure du cou à l´origine d´un défaut de fermeture buccale (A). La patiente a bénéficié d´une chirurgie de réduction à des fins esthétiques et surtout fonctionnelles, c´était initialement sous anesthésie générale puis elle a bénéficié de deux gestes de retouches sous anesthésie locale (B). La patiente a été très satisfaite du résultat avec une fonction restaurée et une esthétique acceptable. Sur un recul de 3 ans, on n´a pas noté de récidive.

**Figure 1:**
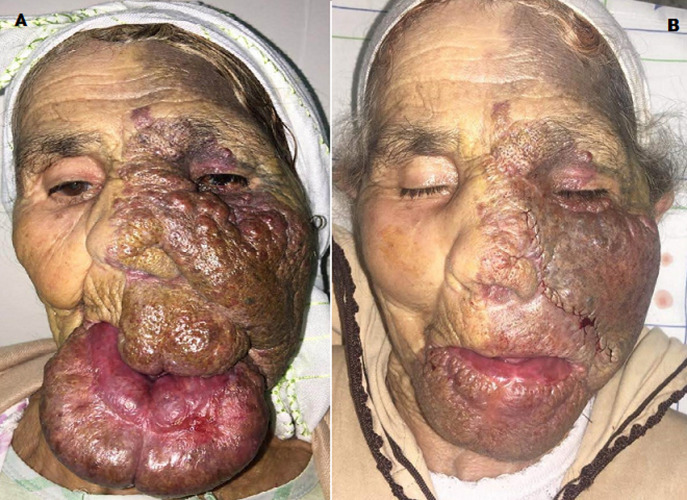
A) énorme angiome de l'hémiface gauche avec une volumineuse lèvre inférieure; B) après la première retouche: résultat satisfaisant

